# Coarser event segmentation and impaired temporal order memory in subclinical ADHD

**DOI:** 10.1007/s00426-026-02274-w

**Published:** 2026-03-10

**Authors:** Carolin Schönenkorb, Sonja A. Kotz, Vincent van de Ven

**Affiliations:** 1 Department of Cognitive Neuroscience, Faculty of Psychology and Neuroscience, Maastricht University, Maastricht, The Netherlands; 2 Department of Neuropsychology & Psychopharmacology, Faculty of Psychology and Neuroscience, Maastricht University, Maastricht, The Netherlands

**Keywords:** Event segmentation, Movie watching, Temporal order memory, ADHD, Dynamic attention theory

## Abstract

We segment a naturalistic, narrative stimulus based on our perception of contextual boundaries. People differ in how they perceive event boundaries, affecting their segmentation and subsequent recall of the temporal structure of the events. Understanding individual difference factors that affect boundary processing will provide further insight in how individuals segment and memorize a naturalistic stimulus. Attention deficit/hyperactivity disorder (ADHD) is a condition that is characterized by altered or impaired temporal attentional allocation. It would follow that participants with a high ADHD predisposition are more likely to miss boundaries and thereby segment a narrative stimulus differently than participants with low ADHD scores. However, it is not known if temporal memory varies with ADHD scores. We report an online study (*N* = 71) in which we tested boundary reporting and temporal order memory performance of a 22-minute sitcom episode in a sample of healthy volunteers that individually varied in self-reported ADHD scores. Results showed a negative correlation between self-reported ADHD scores and number of reported event boundaries, i.e., participants with higher ADHD scores reported fewer boundaries. Further, participants with higher ADHD scores performed more poorly than lower scoring participants for the temporal order of scene frames crossing a boundary, but not for frames presented in the same scene. These findings can be explained by models of impaired dynamic attentional allocation in ADHD. We discuss our findings in light of current literature and suggest that segmentation of naturalistic stimuli is a potentially powerful way of studying cognition and memory in ADHD.

## Introduction

Our daily experiences are fundamentally temporally structured. Making sense of those experiences requires temporal allocation of attention to guide working memory resources and memory formation. For example, when watching a movie, you need to notice the introduction of key characters and temporally track their actions and whereabouts to understand the unravelling of the narrative plotline. There is growing evidence that the brain segments ongoing, temporally structured experiences into discrete mental events, as part of normal perception and memory formation (Baldassano et al., 2017; Heusser et al., 2021; Yeshurun et al., 2017). A key component in this process is the executive control of temporally processing cues on which segmentation is based, such as noticing when a relevant contextual change occurs. Executive function is based on a limited-capacity working memory system, which is involved in clustering information into context-based separate chunks (Baddeley, 2012; Diamond, 2013).

Attention-deficit/hyperactivity-disorder (ADHD) is a neurodevelopmental condition that is often characterized by notable deficits in executive function (Hervey et al., 2004; Kasper et al., 2012; Kofler et al., 2010; Willcutt, 2012, 2023). It has been proposed that deficits in the executive control of temporal attentional allocation is a cardinal feature of ADHD (Castellanos & Tannock, 2002; Sonuga-Barke & Castellanos, 2007; Zheng et al., 2022). Incorrect or inefficient temporal attentional deployment could result in a failure to capture environmental cues signaling a change in context and misguide segmentation of ongoing events, which, in turn, could lead to misinterpreting the meaning of observed actions or events and the formation of event memory. People with (more severe) ADHD could thus show altered segmentation and impaired (working) memory formation of a temporally structured event.

According to event segmentation theories, we store continuous information in discrete events in working memory by inserting event boundaries when perceptual or contextual changes increase and challenge the anticipation of upcoming information (Brunec et al., 2018; Kurby & Zacks, 2008; Radvansky & Zacks, 2017) (but see (Clewett & Davachi, 2017; Güler et al., 2025) for alternative notions). This causes a working memory update and creates a subjective experience of a new event (Kurby & Zacks, 2008; Zacks, 2020). As such, event boundaries support the integration of continuous information into a coherent temporal structure in working memory. Further, boundaries serve as a temporal scaffold for associative memory formation of the segmented experiences (Clewett & Davachi, 2017; Davachi & DuBrow, 2015; Zacks, 2020). For example, participants show enhanced temporal order accuracy for stimuli that were segmented in the same event context during encoding, compared to stimuli that were segmented in different contexts (DuBrow & Davachi, 2013; Heusser et al., 2018; Pu et al., 2022; van de Ven et al., 2022; van de Ven et al., 2023). Event boundaries may thus disrupt ongoing associative processes of temporal relations between sequential stimuli. Boundaries may also guide memory search during retrieval, acting as mnemonic temporal anchors (Michelmann et al., 2023) and biasing recall to items encoded temporally close to boundaries relative to items encoded further away from those boundaries.

However, people differ in how and when they perceive event boundaries, likely impacted by individual working memory differences. For example, working memory was the strongest predictor of how successfully paricipants encoded and segmented short videos of everyday activities (Sargent et al., 2013). More recently, Jafarpour and colleagues (Jafarpour et al., 2022) reported that participants who segmented short movie clips into finer (i.e., identifying more and shorter movie events) or coarser (i.e., identifying fewer and longer events) segments, displayed a faster working memory forgetting rate compared to individuals who showed more normative segmentation. The authors also observed that finer segmentation was accompanied by reduced accuracy on a temporal order task, further suggesting that memory formation depends on how experiences are segmented.

Event segmentation in ADHD has only recently been addressed (Ryan et al., 2023; Ryan & Rogers, 2021). In one study, people clinically diagnosed with ADHD as well as neurotypical controls were asked to segment a short one-minute video-clip and indicate event changes at short and longer time scales (Ryan & Rogers, 2021). Each participant watched a 1-min clip three times with instructions to report any boundaries (Newtson & Engquist, 1976), report boundaries for small events (i.e., fine-grained segmentation) or report boundaries for large events (coarse-grained segmentation). Results showed that ADHD participants reported significantly more boundaries in the coarse-grained condition than neurotypical controls, but that event reporting did not significantly differ between groups in the fine-grained condition. The authors concluded that impaired executive control in people with ADHD increased the frequency of prediction errors over time, resulting in more frequent event updating on longer time scales, although event model updating for shorter time scales was intact. This explanation fits with one interpretation of the model of impaired temporal attentional allocation in ADHD (Castellanos & Tannock, 2002; Mette, 2023), where impaired temporal attention would result in more variable prediction errors and thereby trigger spurious event model updating. However, an alternative interpretation is that temporal attentional deficits in ADHD could lead to coarser (rather than finer) event segmentation if temporal attentional deficits would cause inattentiveness or attentional blindness for contextual changes. For example, if contextual changes are not detected and processed due to limited temporal attentional capacities, boundary detection and event updating would be limited. The latter phenomenon could be partially explained by theories proposing that attention is rhythmically deployed over time (Large & Jones, 1999; Sonuga-Barke & Castellanos, 2007), where attention involuntarily zooms into salient events in a sequence. If so, then this effect may be better measurable when using temporally structured naturalistic stimuli lasting many minutes.

In the present study, we investigated how subclinical ADHD influences the perception of events and subsequent temporal memory reconstruction of a sitcom episode lasting more than 20 min. Healthy participants from an academic environment completed an ADHD symptomatology self-report scale, which were then correlated to performance scores on a segmentation and a temporal memory task. We hypothesized that, if ADHD biases towards more spurious event model updating, i.e., finer segmentation, then ADHD scores should positively correlate with the number of individually reported boundaries. However, if ADHD biases segmentation to coarser segments, we expected a negative correlation between ADHD scores and reported segmentation boundaries. We also hypothesized that ADHD scores would be negatively correlated with temporal order memory performance.

## Methods

### Participants

Seventy-one (*N* = 71) healthy volunteers (mean (SD) age in years = 21.20 (2.54), range = 17–28) completed the experiment online. An a priori power calculation (two-tailed, effect size = 0.35, $\alpha$= 0.05, $1-\beta$=0.80) suggested a minimum sample size of 59 participants. Participants were recruited from the academic community of Maastricht University via the SONA recruitment system, social media, or personal contact. Participants received a financial compensation or equivalent course credit. Participants with a poor or unstable internet connection that prevented them from starting or completing the online session (see below) were excluded from the experiment. Online informed consent was provided by all participants prior to starting the experiment. The ethical review board of the Faculty of Psychology and Neuroscience approved the study (OZL 231_139_12_2020_S2). The study was not previously preregistered.

### Procedures

The study was conducted online via Pavlovia, which is part of PsychoPy (Bridges et al., 2020; Peirce et al., 2019). Prior to starting the online experiment, participants received detailed instructions and completed a headphone test to ensure they wore headphones to minimize distractions (Woods et al., 2017). The headphone test included six trials, in each of which participants had to judge which of three pure tones had to lowest volume, with one of the tones presented 180° out of phase across the stereo channels. Participants received feedback on the correctness of their judgment for each trial and continued with the experiment if at least five trials were answered correctly.

To investigate the effects of subclinical ADHD on event segmentation and temporal memory for a narrative stimulus, we administered the 18-items Adult ADHD self-report scale (ASRS) v1.1 Symptom Checklist ((Kessler et al., 2005); retrieved from https://www.hcp.med.harvard.edu/ncs/asrs.php). The self-report scale is based on the fourth version of the Diagnostic and Statistical Manual of Mental Disorders (DSM–4; American Psychiatric Association, 1994). Adult participants with a total score higher than 24 are considered to likely have ADHD. For each item, participants must indicate how frequently they experience a described behavior or thought (e.g., “How often do you have problems remembering appointments or obligations?”) using a 5-point rating scale (0 = Never to 4 = Very often). The ASRS shows overall good internal consistency (*α* = 0.84 Adler et al., 2006) for affected persons and good inter-rater reliability (ICC=0.83 Adler et al., 2006). We used the ASRS total score as a continuous variable to investigate its relationship with event segmentation and temporal memory.

Before the experiment started, participants completed a short practice trial, which included a short movie clip (2 min) that they had to segment (“press the SPACEBAR when one natural and meaningful unit ends, and another begins” (Newtson & Engquist, 1976; Schwan & Garsoffky, 2004)). Then, they completed a short memory task related to the video. After the practice trial, participants completed the main experiment, where they watched an episode of the British comedic sitcom *Black Books* (episode 1, season 1, *Cooking the books*, duration 22 min 21 s). The episode introduces the main protagonist of the series, grumpy and foul-tempered bookstore owner Bernard Black, and his staff trying to cope with the protagonist’s erratic behavior, while his dodgy accountant goes on the run from the police. The series is critically acclaimed and has received high viewer ratings and positive reviewing of its depiction of “British humor” (e.g., 88% critics score on Rotten Tomatoes; 8.7 out of 10 on Metacritic). The video was presented in the center of a computer screen. Participants received the same segmentation instructions as for the practice trial.

Following the movie segmentation, participants completed a temporal order memory task, in which they saw trials of two still frames from the previously segmented episode. They had to indicate which of the two frames was presented first during the episode by pressing the corresponding keyboard key (“1”=left frame was shown first, “2”=right frame was shown first). We selected frame pairs from consecutive scenes (across-scenes trials), in which the pairs crossed a scene boundary, or from the same scene (within-scene trials). Prototypical boundaries were obtained from a different study (Schoenenkorb et al., 2023). In brief, an independent sample of participants (N = 10, age range = 22–25 years) viewed the episode with the instructions to mark event boundaries similar as in the current study. We then averaged timestamps of the button presses that occurred within a 10 s window across participants. Timestamp averages of six or more participants out of ten were labeled as reliable (i.e., high-agreement) boundaries. Finally, one of the experimenters (author CS) identified the start of a scene related to each boundary at a high temporal precision (in 100 msec resolution), which we refer to as “prototypical boundaries”. For the temporal memory task, frame pairs were selected with a temporal distance of 30 seconds between the frames. The complete stimulus set included 14 across-scene frame pairs and 14 same-scene pairs. The earlier frame of a pair was presented left of the screen in half of the trials and on the right in the other half. Trial order was randomized for each participant and there was no response time limit.

**Fig. 1:**
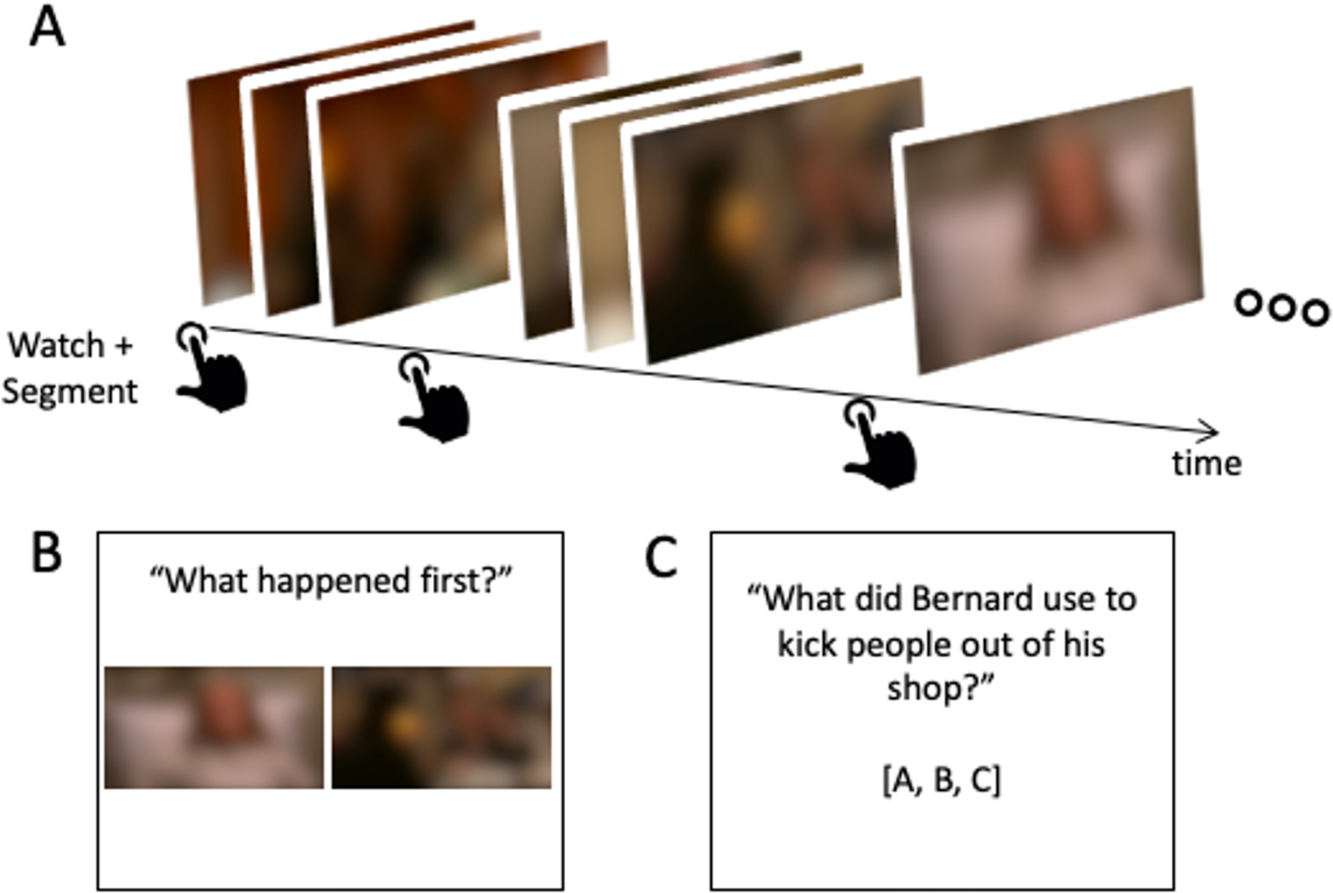
Experimental design. Participants watched and segmented a sitcom episode (**A**) and then completed a temporal order memory task (**B**) and a semantic knowledge task with 3-option multiple choice questions (**C**)

Finally, to test how much participants were engaged with the viewing task and remembered relevant narrative details, participants were tested regarding their semantic knowledge of the episode. In each of fourteen trials, participants were presented with a question about a narrative element of the episode and had to select the correct answer from three options (Fig. 1C). The order of questions was randomized for each participant. Their response time was unlimited.

### Analysis

Statistical analysis was performed in JASP (version 0.17.2) and Matlab. For the ASRS scores, we calculated the internal consistency of the scale using Cronbach’s alpha. For the movie segmentation task, we analysed the frequency of segmentation responses as the total number of button presses (irrespective of response timing). To analyse the timing of boundary responses, we created temporal vectors for each participant at a resolution of 100 msec for the full duration of the episode (resulting in 13,200 timepoints per vector), in which the timepoint associated to a boundary response was set to 1 and all other timepoints to 0. To analyse the timing of idiosyncratic boundary responses relative to the prototypical boundaries, we calculated lagged correlations between the temporal vectors of each participant and the vector of the prototypical boundaries. We then calculated the degree of agreement between an individual’s temporal vector (idiosyncratic segmentation) and the vector representing the independently identified boundaries (prototypical segmentation), using the Jaccard index of overlap between binary response vectors, *A* and *B*, such that

$$J(A,B)=\frac{\left|A\cap B\right|}{\left|A\cup B\right|}$$

with the numerator indicating the number of 1 s overlapping in time between both vectors and the denominator indicating all 1 s in either set. To correct for inter-individual variation in response timing for similar event boundaries, we superimposed a 5 s window (0.38% of episode duration) of 1 s centered on each singular response timepoint. This is comparable to Gaussian convolution of $\sigma=2$ (approximating full-width-at-half-maximum (FWHM) = 5 s) but retaining a discretized coding of 0 s and 1s. A higher Jaccard index value indicates a higher overlap between idiosyncratic and prototypical boundaries.

We also calculated a more commonly used metric of similarity between boundary responses across individuals (Michelmann et al., 2021; Sasmita & Swallow, 2023; Zacks, Tversky et al., 2001b). For this approach, the temporal vectors of singular boundary responses were convolved using a Gaussian kernel ($\sigma=2$), after which each convolved vector was compared to a leave-one-out group average of all other participants’ vectors using Pearson correlation. Notably, this metric represents boundary alignment across participants, whereas the Jaccard index captures each participant’s overlap of reported boundaries with a prototypical set of boundaries. (We note here that the width of the convolution is a free parameter. We ran the same analyses reported below with different convolution parameters ranging between 0.5 and 2 and found qualitatively similar results.)

For the temporal order memory task, we calculated the average hit rate of temporal order judgments for the within-scene and the across-scene conditions. Trials with response times shorter than 500 msec or longer than 15 s were discarded (2.78% of all trials). We used paired sample t-tests to compare the performance of the two conditions. For the semantic knowledge test, we calculated proportion correct by dividing the sum of correctly answered trials by the total number of trials. Chance performance was 0.33. To investigate the relation between ASRS scores and task performance, we calculated Pearson correlations between ASRS scores and segmentation performance (number of segmentations, Jaccard index values and agreement scores) and memory performance (temporal order memory and semantic memory scores). The significance threshold was a priori set to alpha = 0.05.

## Results

### ASRS scores

Cronbach’s alpha for the ASRS questionnaire was 0.83, which indicates high internal consistency and aligns well to previous findings (Adler et al., 2006; Green et al., 2019; Kessler et al., 2005). The distribution of ASRS scores ranged between 12 and 62, with a mean (SD) score of 29.14 (8.77) and a 95% confidence interval of [27.06, 31.22]. The median ASRS score was 28. The majority of participants showed scores in the range of highly likely to have ADHD ((Kessler et al., 2005); *N* = 41, 64.8%).

### Segmentation

**Fig. 2:**
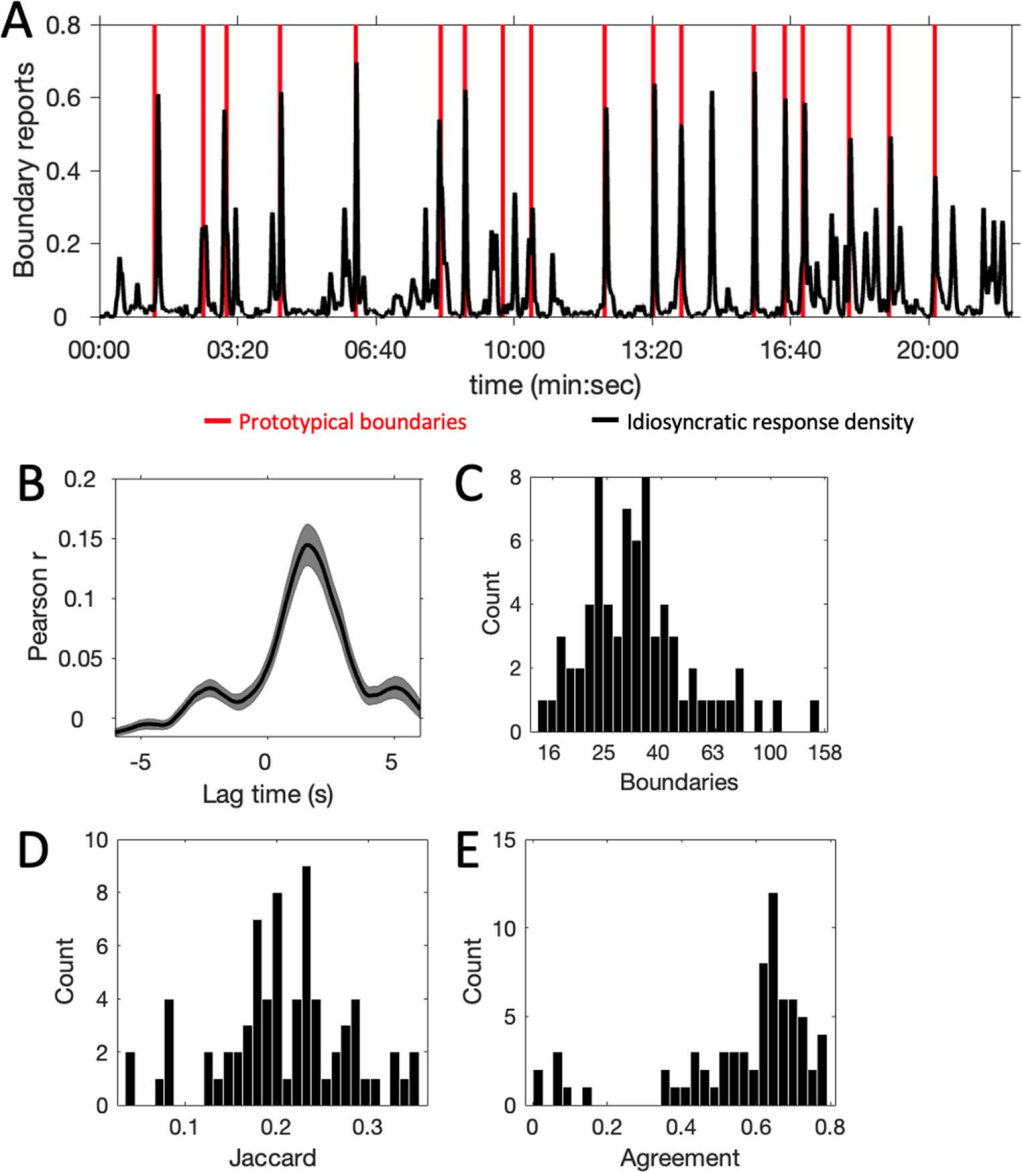
Segmentation results. **A**: Averaged idiosyncratic boundary reports (black line) as a function of episode time. Red vertical lines indicate prototypical boundaries. **B**: Lagged correlation between the prototypical and idiosyncratic response vectors. The rightward displacement of the peak of the curve indicates that boundary responses occurred after the prototypical boundaries. Shaded area indicates 95% confidence interval. **C**, **D**,**E**: Frequency distributions of number of idiosyncratic boundaries (**C**), Jaccard similarity index (**D**) and the inter-subject agreement score (**E**)

Figure 2A shows the averaged segmentation response vectors (black line) in relation to the prototypical boundaries (red vertical lines). On average, participants’ segmentation responses tended to occur shortly after the prototypical boundaries, in line with previous reports of solid participant agreement for “strong” narrative boundaries (Huff et al., 2014; Sargent et al., 2013). The lagged correlation analysis confirmed the delayed timing of boundary responses with respect to the prototypical boundaries (Fig. 2B), with a significantly delayed mean peak latency (mean (SE) peak time = 1.19 (0.15) seconds, 95% CI=[0.89, 1.49], t(70) = 7.89, *p* < 0.001, Cohen’s d = 0.94).

There was also substantial variation in segmentation reporting over the course of the episode. Figure 2C shows the distribution of the number of reported idiosyncratic boundaries, which ranged between 14 and 151 (mean (SD) = 36.80 (22.05), median = 32, 95% CI=[31.58, 42.02]). Figure 2D shows the distribution of Jaccard index values indicating the overlap between the idiosyncratic and prototypical segmentations, which ranged between 0.03 and 0.36 (mean (SD) = 0.21 (0.07), median = 0.20, 95% CI=[0.19, 0.23]). The number of segmentations did not significantly correlate with the Jaccard similarity index (*r*=−0.21, *p* = 0.08). The inter-subject agreement score showed overall good agreement (Fig. 2E; mean (SD) = 0.56 (0.20), 95% CI=[0.52, 0.61]). The agreement score correlated significantly with the Jaccard index (r(69) = 0.85, *p* < 0.001), suggesting that average pattern of idiosyncratic boundary detection was similar to the pattern of prototypical boundaries. Agreement scores did not correlate significantly with boundary response frequency (*r*=−0.01, *p* = 0.92).

Correlation analysis between ASRS scores and number of segmentation responses revealed a significant negative correlation (r(69)=−0.26, *p* = 0.026), with number of segmentation responses decreasing with higher ASRS scores. The ASRS scores did not significantly correlate with Jaccard indexes (r(69)=−0.08, *p* = 0.50) or agreement scores (r(69)=−0.11, *p*=−0.35), indicating that idiosyncratic similarity to prototypical segmentation did not vary with ASRS scores.

### Temporal order memory task

**Fig. 3:**
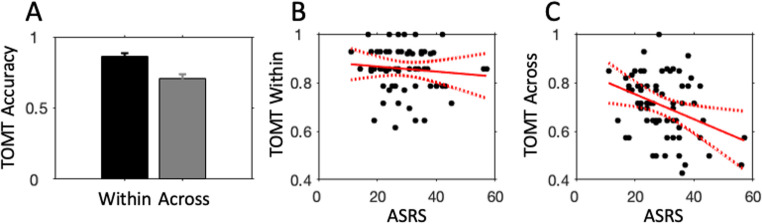
Temporal order memory results. **A**: Average within-scene (black bar) and across-scenes (gray bar) temporal order memory accuracy. Error bars indicate 95% confidence intervals. **B**, **C**: Scatter plots between ASRS scores and within-scene (**B**) and across-scenes temporal order accuracy (**C**). Red lines indicate linear trend (solid line) and 95% confidence intervals (dotted lines)

Figure 3A shows average temporal order memory accuracy for the within-scene and across-scenes conditions. Participants significantly performed better (t(69) = 9.16, *p* < 0.001, Cohen’s d = 1.09) in the within-scene condition (mean (SE) = 0.86 (0.01) than in the across-scenes condition (mean (SE) = 0.71 (0.02). This finding is in line with previous findings of superior within-event compared to across-events performance (DuBrow & Davachi, 2013; Heusser et al., 2018; Pu et al., 2022; van de Ven et al., 2023).

To test if ASRS scores predict overall temporal order memory performance, we correlated ASRS scores with the average of the two temporal order task conditions. ASRS scores significantly negatively correlated with overall temporal memory performance (r(69)=−0.32, *p* = 0.007), suggesting worse temporal order accuracy with higher ASRS scores. We then tested if this association was related to the role of boundaries in temporal order memory, as segmentation contributes differently to within- and across-events temporal order memory. We correlated ASRS scores with the difference between the within- and across-scenes values and found a significant positive correlation (r(69) = 0.25, *p* = 0.034) of higher ASRS scores with larger boundary-based temporal memory difference. We then correlated ASRS scores with performance in each memory condition. ASRS scores did not significantly correlate with within-scene temporal order accuracy (Fig. 3B; r(69)=−0.10, *p* = 0.40), but did significantly negatively correlate with across-scenes accuracy (Fig. 3C; r(69)=−0.36, *p* = 0.002), in which across-scenes temporal order performance decreased with increasing ASRS scores. That is, the temporal order memory that is commonly found to decrease after boundary processing showed a further decrement in participants with higher ASRS scores. In sum, these findings suggest that boundary-based temporal order memory performance is more impaired in persons with higher subclinical ADHD scores, while within-scene temporal memory is spared.

We also tested if temporal order memory accuracy was related to segmentation performance (see Table 1). Memory accuracy, averaged across the two conditions, did not significantly correlate with the number of boundaries. There was a close-to-significant correlation with the Jaccard indexes and a significant correlation with the agreement scores. Both metrics of boundary alignment correlated significantly with across-events order memory but not with within-events order memory.

**Table 1:** Temporal order memory correlations with segmentation

Condition	Nsegm	Jaccard	Agreement
All	0.04	0.23^a^	0.32**
Within	−0.07	0.04	0.16
Across	0.11	0.28*	0.32**

Pearson correlation values (degrees of freedom = 69) are listed for the average of the within- and across-events conditions (“All”) and for each condition separately with number of segmentations (Nsegm), the Jaccard indexes and the agreement scores. a *p* = 0.057, * *p* < 0.05, ** *p* < 0.01

### Semantic memory

Participants showed a relatively high semantic memory performance (mean (SD) = 0.88 (0.11), 95% CI=[0.85, 0.91]), which was well above chance (33%, *p* < 0.001) but did not indicate ceiling performance. Participants were thus generally engaged enough during episode watching to remember narrative knowledge. Semantic memory accuracy correlated with Jaccard indexes (r(69) = 0.23, *p* = 0.051) and agreement scores (r(69) = 0.34, *p* = 0.002), indicating a relation with how much idiosyncratic boundaries aligned to the prototypical boundaries. Semantic memory accuracy did not correlate with ASRS scores (r(69)=−0.11, *p* = 0.34) or with the number of idiosyncratic boundaries (r(69) = 0.08, 0.53), suggesting that participants learned narrative knowledge regardless of ASRS scores. Semantic memory accuracy correlated significantly with overall temporal memory accuracy (pooling within- and across-events conditions, r(69) = 0.35, *p* = 0.003), indicating a general memory effect across the memory tasks.

## Discussion

In this study, we investigated the effects of subclinical ADHD ratings on event encoding and temporal memory. We found two main results. First, participants with higher subclinical ADHD scores reported fewer event boundaries. This finding seems to contrast the findings reported by Ryan and Rogers (Ryan & Rogers, 2021), who found that individuals clinically diagnosed with ADHD segmented more events than their controls. However, in their study, ADHD participants tended to report more segmentation for long events, but fewer short events and overall fewer segmentation responses, although this difference was not statistically significant. A direct comparison between their study and ours is made difficult due to the different methodologies used, such as the much shorter videos being presented multiple times versus our one-time viewing of a longer narrative sitcom episode. Our findings do not match the suggestion that attentional or working memory deficits in ADHD would result in more frequent prediction errors biasing towards fine-grain segmentation, which would predict the reporting of more, not fewer boundaries.

One consideration is that participants with high ASRS scores could have had more difficulty executing the dual task nature of a segmentation task, which required them to retain engagement throughout the episode while also overtly reporting event boundaries by button pressing. That is, participants with high ASRS scores could forget to press the button. This scenario cannot be fully ruled out from our study design. However, we argue that our findings provide at least some support for the notion of ADHD being related to detecting fewer, not more boundaries. The number of boundary reports did not correlate with the inter-subject agreement of idiosyncratic boundaries or with the agreement to the prototypical boundaries, indicating that participants generally tended to report the “strong” or reliable boundaries. High ASRS participants thus seem to have underreported less obvious or agreeable contextual changes, which does not align with an overall forgetting to press the button.

Second, we also found less accurate across-scene temporal order performance in high ASRS participants. This effect did not correlate with the sparser boundary reporting observed for high ASRS participants, suggesting different underlying mechanisms for boundary detection and boundary-based event processing in memory. The impaired temporal order performance cannot be simply explained by poor overall memory formation due to inattentiveness. The high semantic memory performance, in combination with the high within-scene temporal order accuracy, suggests that participants viewed the episode attentively enough to learn movie knowledge. Further, the segmentation instructions combined with a strongly engaging narrative stimulus arguably facilitated attentional performance, in which case the accuracy decrement in high ASRS participants is even more remarkable. This effect is also unlikely to stem from a general deficit in associative memory formation in ADHD, independent of attentional encoding or executive functioning, for which the literature reports weak to no evidence (Kofler et al., 2018; Skodzik et al., 2017; Skowronek et al., 2008). Instead, our finding suggests an impairment in boundary-based event memory formation or reconstruction in ADHD.

In all, our findings fit very well to the notion of working memory deficits in ADHD. From the viewpoint of dynamic attentional theories (Large & Jones, 1999; Sonuga-Barke & Castellanos, 2007), ADHD participants could be less attentive to contextual changes, and therefore would not update their event models in working memory. That is, higher (sub-)clinical ADHD scores could lead to contextual change blindness, resulting in failure to detect event boundaries and thereby affect temporal segmentation. Smaller perceptual changes would be more likely to go undetected than more salient conceptual or higher-order changes in the narrative. It is also possible that working memory deficits reside in event model updating, such as the flushing or updating of previous event models from working memory when encountering an event boundary (Güler et al., 2024; Ongchoco & Scholl, 2019). There is ample evidence for a deficit in working memory updating or content control in ADHD (Keage et al., 2008; Marchetta et al., 2008; Zhao et al., 2020), which play a role in event memory flushing. In boundary-based segmentation, such impairments could disrupt the temporal associations between events, amplifying the cost of boundary processing on temporal binding between events (Heusser et al., 2018), but leave temporal binding within an event intact. Impaired boundary processing in working memory could also further tax temporal processing and cognition that are already compromised in ADHD (Castellanos & Tannock, 2002; Mette, 2023; Zheng et al., 2022). Interestingly, a growing number of studies have challenged the notion that event segmentation depends on the detection of contextual change or event model prediction error (Clewett & Davachi, 2017; Güler et al., 2025), which opens up the possibility that segmentation impairments in ADHD may encompass other working memory mechanisms as well. To further parse these mechanisms, future studies could investigate the neural mechanisms associated to boundary detection or processing impairments in ADHD. For example, event segmentation is considered an automatic process that occurs regardless of whether participants overtly report boundaries or not (Chen et al. 2017; Zacks 2020; Zacks et al. 2001a, b). Investigating neural responses to event boundaries in the absence of overt boundary responding (e.g., (Baldassano et al., 2017; Chen et al., 2017; Ezzyat & Clements, 2024) could then be used to test if and how boundary processing differs between ADHD and neurotypical participants.

Our study is the first to use a narrative stimulus in the context of segmentation and temporal memory formation in subclinical ADHD. Previous studies focused on segmentation performance using shorter clips of 1 to several minutes in duration (Ryan et al., 2023; Ryan & Rogers, 2021). Our approach fits with current developments to study healthy and pathological cognition using naturalistic stimuli and in more real-life settings (Kurby et al., 2014; Richmond et al., 2017; Willems et al., 2020). One advantage of using sitcoms or movies is that these stimuli are designed to draw attention from the viewer during watching, which in the study of ADHD or ADHD-like syndromes can be advantageous for attention on a task. This juxtaposes the more experientially tedious, but also more experimentally controllable artificial lab design that allows more careful testing of isolated experimental factors. Additionally, movies induce schema-based prior knowledge (Bower & Morrow, 1990; Frisoni et al., 2021, 2022) that is absent for more controlled experimental designs in which stimuli are randomly selected. It is not known if schema memory varies with ADHD-related symptomatology. However, comparable segmentation and temporal memory results have been obtained in healthy participants with a number of different encoding formats using pictures (DuBrow & Davachi, 2014; Heusser et al., 2018; van de Ven et al., 2022), virtual reality (Logie & Donaldson, 2021; van Helvoort et al., 2020), short video clips (Hard et al., 2006; Sargent et al., 2013) and audio stimuli (Lositsky et al., 2016; Michelmann et al., 2021; van de Ven et al., 2023). Hence, we argue that our reported effects are likely to generalize to other segmentation paradigms. We suggest that a combination of both methodologies (i.e., naturalistic and experimentally controlled), possibly together with advanced analysis tools, are a powerful way forward in studying cognition and behavior in atypical populations.

Finally, we note some limitations of the current experiment and results. First, we administered the experimental design online, which made it difficult to verify if participants were indeed attentive during encoding and put effort into the retrieval tasks. The online environment may be exceptionally challenging for participants with a high (subclinical) disposition to ADHD. To be sure, we argue that the use of an entertaining narrative stimulus and the instructions to wear headphones to limit environmental distractions mitigated the risk of inattentiveness, and the high accuracy for within-scene temporal order judgments and semantic knowledge further suggests that participants attended the stimulus enough to remember relevant story elements. However, we cannot rule out the possibility of limited attentive compliance in an online environment. Second, we did not include an empirical measure of executive functioning, which would be necessary to assess in how far these results are directly related to degrees of cognitive impairment across the ADHD symptomatology spectrum. Third, our sample comprised of young academic students who very likely show a high-functioning range of participants predisposed to ADHD. The generalizability of these findings to the general population or to clinical ADHD thus remains to be tested. Fourth, our prototypical boundaries, which reflected high inter-subject agreement, did not allow us to investigate idiosyncratic boundaries with low inter-subject agreement. Low agreement boundaries may be related to fine-grained segmentation that is based on smaller perceptual or narrative changes. There is evidence that fine- and coarse-grained boundaries may be related to different working memory and neural mechanisms (Jafarpour et al., 2022), which may limit generalization of our findings.

In conclusion, we show that increased subclinical ADHD scores are associated with decreased boundary reporting and impaired temporal order judgments for contents crossing event boundaries in memory. We suggest that these effects arise from inattentiveness or attentional blindness to contextual changes, which affect segmentation and subsequently temporal memory formation. We suggest that it may be feasible to use relatively long-lasting naturalistic stimuli to study cognition and memory in sub-clinical as well as clinical groups.

## Data Availability

All behavioral data and study materials are available on an Open Science Foundation page (10.17605/OSF.IO/SZ2AG).

## Declarations

### Generative AI and AI-assisted technologies in the writing process

No generative AI or AI-assisted technologies were used in the writing or editing of this manuscript or in the creation of the presented figures.

## References

[CR1] Adler, L. A., Spencer, T., Faraone, S. V., Kessler, R. C., Howes, M. J., Biederman, J., & Secnik, K. (2006). Validity of pilot adult ADHD Self-Report Scale (ASRS) to rate adult ADHD symptoms. *Annals of Clinical Psychiatry,**18*(3), 145–148. 10.1080/10401230600801077 16923651 10.1080/10401230600801077

[CR2] Baddeley, A. D. (2012). Working memory: Theories, Models, and Controversies. *Annual Review of Psychology,**63*, 1–29. 10.1146/annurev-psych-120710-100422 21961947 10.1146/annurev-psych-120710-100422

[CR3] Baldassano, C., Chen, J., Zadbood, A., Pillow, J. W., Hasson, U., & Norman, K. A. (2017). Discovering event structure in continuous narrative perception and memory. *Neuron,**95*(3), 709-721.e5. 10.1016/j.neuron.2017.06.041 28772125 10.1016/j.neuron.2017.06.041 PMC5558154

[CR4] Bower, G. H., & Morrow, D. G. (1990). Mental models in narrative comprehension. *Science,**247*(4938), 44–48. 10.1126/science.2403694 2403694 10.1126/science.2403694

[CR5] Bridges, D., Pitiot, A., MacAskill, M. R., & Peirce, J. W. (2020). The timing mega-study: Comparing a range of experiment generators, both lab-based and online. *PeerJ,**8*, Article e9414. 10.7717/peerj.9414 33005482 10.7717/peerj.9414 PMC7512138

[CR6] Brunec, I. K., Moscovitch, M., & Barense, M. D. (2018). Boundaries shape cognitive representations of spaces and events. *Trends in Cognitive Sciences,**22*(7), 637–650. 10.1016/j.tics.2018.03.013 29706557 10.1016/j.tics.2018.03.013

[CR7] Castellanos, F. X., & Tannock, R. (2002). Neuroscience of attention-deficit/hyperactivity disorder: The search for endophenotypes. *Nature Reviews Neuroscience,**3*(8), 617–628. 10.1038/nrn896 12154363 10.1038/nrn896

[CR8] Chen, J., Leong, Y. C., Honey, C. J., Yong, C. H., Norman, K. A., & Hasson, U. (2017). Shared memories reveal shared structure in neural activity across individuals. *Nature Neuroscience*, *20*(1), 115–125. 10.1038/nn.4450 27918531 10.1038/nn.4450 PMC5191958

[CR9] Clewett, D., & Davachi, L. (2017). The ebb and flow of experience determines the temporal structure of memory. *Current Opinion in Behavioral Sciences*, *17*, 186–193. 10.1016/j.cobeha.2017.08.013 29276730 10.1016/j.cobeha.2017.08.013 PMC5739077

[CR10] Davachi, L., & DuBrow, S. (2015). How the hippocampus preserves order: The role of prediction and context. *Trends in Cognitive Sciences*, *19*(2), 92–99. 10.1016/j.tics.2014.12.004 25600586 10.1016/j.tics.2014.12.004 PMC4380862

[CR11] Diamond, A. (2013). Executive functions. *Annual Review of Psychology*, *64*, 135–168. 10.1146/annurev-psych-113011-143750 23020641 10.1146/annurev-psych-113011-143750 PMC4084861

[CR12] DuBrow, S., & Davachi, L. (2013). The influence of context boundaries on memory for the sequential order of events. *Journal of Experimental Psychology: General*, *142*(4), 1277–1286. 10.1037/a0034024 23957281 10.1037/a0034024 PMC3902141

[CR13] DuBrow, S., & Davachi, L. (2014). Temporal memory is shaped by encoding stability and intervening item reactivation. *Journal of Neuroscience,**34*(42), 13998–14005. 10.1523/JNEUROSCI.2535-14.2014 25319696 10.1523/JNEUROSCI.2535-14.2014 PMC4198540

[CR14] Ezzyat, Y., & Clements, A. (2024). Neural activity differentiates novel and learned event boundaries. *Journal of Neuroscience,**44*(38), 1–13. 10.1523/JNEUROSCI.2246-23.2024 10.1523/JNEUROSCI.2246-23.2024 PMC11411582 38871462

[CR15] Frisoni, M., Di Ghionno, M., Guidotti, R., Tosoni, A., & Sestieri, C. (2021). Reconstructive nature of temporal memory for movie scenes. *Cognition,**208*(April 2020), Article 104557. 10.1016/j.cognition.2020.104557 33373938 10.1016/j.cognition.2020.104557

[CR16] Frisoni, M., Di Ghionno, M., Guidotti, R., Tosoni, A., & Sestieri, C. (2022). Effects of a narrative template on memory for the time of movie scenes: Automatic reshaping is independent of consolidation. *Psychological Research,**87*(2), 598–612. 10.1007/s00426-022-01684-w 35524807 10.1007/s00426-022-01684-w PMC9076810

[CR17] Green, J. G., DeYoung, G., Wogan, M. E., Wolf, E. J., Lane, K. L., & Adler, L. A. (2019). Evidence for the reliability and preliminary validity of the Adult ADHD Self-Report Scale v1.1 (ASRS v1.1) Screener in an adolescent community sample. *International Journal of Methods in Psychiatric Research,**28*(1), Article 6. 10.1002/mpr.1751 10.1002/mpr.1751 PMC6877133 30407687

[CR18] Güler, B., Adıgüzel, Z., Uysal, B., & Günseli, E. (2024). Discrete memories of a continuous world: A working memory perspective on event segmentation. *Current Research in Behavioral Sciences,**6*, Article 100145. 10.1016/j.crbeha.2023.100145

[CR19] Güler, B., Serin, F., & Günseli, E. (2025). Prediction error is out of context: The dominance of contextual stability in structuring episodic memories. *Psychonomic Bulletin & Review*(6). 10.3758/s13423-025-02723-4 10.3758/s13423-025-02723-4 PMC12627157 40571834

[CR20] Hard, B. M., Tversky, B., & Lang, D. S. (2006). Making sense of abstract events: Building event schemas. *Memory & Cognition,**34*(6), 1221–1235. 10.3758/BF03193267 17225504 10.3758/bf03193267

[CR21] Hervey, A. S., Epstein, J. N., & Curry, J. F. (2004). Neuropsychology of adults with ADHD a meta-analytic review. *Neuropsychology,**18*(3), 485–503. 15291727 10.1037/0894-4105.18.3.485

[CR22] Heusser, A. C., Ezzyat, Y., Shiff, I., & Davachi, L. (2018). Perceptual boundaries cause mnemonic trade-offs between local boundary processing and across-trial associative binding. *Journal of Experimental Psychology. Learning, Memory, and Cognition,**44*(7), 1075–1090. 10.1037/xlm0000503 29461067 10.1037/xlm0000503 PMC6013306

[CR23] Heusser, A. C., Fitzpatrick, P. C., & Manning, J. R. (2021). Geometric models reveal behavioural and neural signatures of transforming experiences into memories. *Nature Human Behaviour,**5*(7), 905–919. 10.1038/s41562-021-01051-6 33574605 10.1038/s41562-021-01051-6

[CR24] Huff, M., Meitz, T. G. K., & Papenmeier, F. (2014). Changes in situation models modulate processes of event perception in audiovisual narratives. *Journal of Experimental Psychology: Learning, Memory, and Cognition,**40*(5), 1377–1388. 10.1037/a0036780 24820670 10.1037/a0036780

[CR25] Jafarpour, A., Buffalo, E. A., Knight, R. T., & Collins, A. G. E. (2022). Event segmentation reveals working memory forgetting rate. *iScience*. 10.1016/j.isci.2022.103902 35252809 10.1016/j.isci.2022.103902 PMC8891967

[CR26] Kasper, L. J., Alderson, R. M., & Hudec, K. L. (2012). Moderators of working memory deficits in children with attention-deficit/hyperactivity disorder (ADHD): A meta-analytic review. *Clinical Psychology Review,**32*(7), 605–617. 10.1016/j.cpr.2012.07.001 22917740 10.1016/j.cpr.2012.07.001

[CR27] Keage, H. A. D., Clark, C. R., Hermens, D. F., Williams, L. M., Kohn, M. R., Clarke, S., Lamb, C., Crewther, D., & Gordon, E. (2008). ERP indices of working memory updating in AD/HD: Differential aspects of development, subtype, and medication. *Journal of Clinical Neurophysiology,**25*(1), 32–41. 10.1097/WNP.0b013e318163ccc0 18303558 10.1097/WNP.0b013e318163ccc0

[CR28] Kessler, R. C., Adler, L., Ames, M., Demler, O., Faraone, S., Hiripi, E., Howes, M. J., Jin, R., Secnik, K., Spencer, T., Ustun, T. B., & Walters, E. E. (2005). The World Health Organization adult ADHD self-report scale (ASRS): A short screening scale for use in the general population. *Psychological Medicine*, *35*(2), 245–256. 10.1017/S0033291704002892 15841682 10.1017/s0033291704002892

[CR29] Kofler, M. J., Rapport, M. D., Bolden, J., Sarver, D. E., & Raiker, J. S. (2010). ADHD and working memory: The impact of central executive deficits and exceeding storage/rehearsal capacity on observed inattentive behavior. *Journal of Abnormal Child Psychology,**38*(2), 149–161. 10.1007/s10802-009-9357-6 19787447 10.1007/s10802-009-9357-6

[CR30] Kofler, M. J., Spiegel, J. A., Austin, K. E., Irwin, L. N., Soto, E. F., & Sarver, D. E. (2018). Are episodic buffer processes intact in ADHD? Experimental evidence and linkage with hyperactive behavior. *Journal of Abnormal Child Psychology,**46*(6), 1171–1185. 10.1007/s10802-017-0346-x 28952051 10.1007/s10802-017-0346-x PMC5871530

[CR31] Kurby, C. A., & Zacks, J. M. (2008). Segmentation in the perception and memory of events. *Trends in Cognitive Sciences,**12*(2), 72–79. 10.1016/j.tics.2007.11.004 18178125 10.1016/j.tics.2007.11.004 PMC2263140

[CR32] Kurby, C. A., Asiala, L. K. E., & Mills, S. R. (2014). Aging and the segmentation of narrative film. *Aging, Neuropsychology, and Cognition,**21*(4), 444–463. 10.1080/13825585.2013.832138 10.1080/13825585.2013.832138 23984846

[CR33] Large, E. W., & Jones, M. R. (1999). The dynamics of attending: How people track time-varying events. *Psychological Review,**106*(1), 119–159. 10.1037/0033-295X.106.1.119

[CR34] Logie, M. R., & Donaldson, D. I. (2021). Do doorways really matter: Investigating memory benefits of event segmentation in a virtual learning environment. *Cognition,**209*(January), Article 104578. 10.1016/j.cognition.2020.104578 33422863 10.1016/j.cognition.2020.104578

[CR35] Lositsky, O., Chen, J., Toker, D., Honey, C. J., Shvartsman, M., Poppenk, J. L., Hasson, U., & Norman, K. A. (2016). Neural pattern change during encoding of a narrative predicts retrospective duration estimates. *eLife*. 10.7554/eLife.16070 27801645 10.7554/eLife.16070 PMC5243117

[CR36] Marchetta, N. D. J., Hurks, P. P. M., Krabbendam, L., & Jolles, J. (2008). Interference control, working memory, concept shifting, and verbal fluency in adults with Attention-Deficit/Hyperactivity Disorder (ADHD). *Neuropsychology,**22*(1), 74–84. 10.1037/0894-4105.22.1.74 18211157 10.1037/0894-4105.22.1.74

[CR37] Mette, C. (2023). Time perception in adult ADHD: Findings from a decade—A review. *International Journal of Environmental Research and Public Health*. 10.3390/ijerph20043098 36833791 10.3390/ijerph20043098 PMC9962130

[CR38] Michelmann, S., Price, A. R., Aubrey, B., Strauss, C. K., Doyle, W. K., Friedman, D., Dugan, P. C., Devinsky, O., Devore, S., Flinker, A., Hasson, U., & Norman, K. A. (2021). Moment-by-moment tracking of naturalistic learning and its underlying hippocampo-cortical interactions. *Nature Communications*. 10.1038/s41467-021-25376-y 34518520 10.1038/s41467-021-25376-y PMC8438040

[CR39] Michelmann, S., Hasson, U., & Norman, K. A. (2023). Evidence that event boundaries are access points for memory retrieval. *Psychological Science,**34*(3), 326–344. 10.1177/09567976221128206 36595492 10.1177/09567976221128206 PMC10152118

[CR40] Newtson, D., & Engquist, G. (1976). The perceptual organization of ongoing behavior. *Journal of Experimental Social Psychology,**12*(5), 436–450. 10.1016/0022-1031(76)90076-7

[CR41] Ongchoco, J. D. K., & Scholl, B. J. (2019). Did that just happen? Event segmentation influences enumeration and working memory for simple overlapping visual events. *Cognition,**187*, 188–197. 10.1016/j.cognition.2019.01.002 30897509 10.1016/j.cognition.2019.01.002

[CR42] Peirce, J., Gray, J. R., Simpson, S., MacAskill, M., Höchenberger, R., Sogo, H., Kastman, E., & Lindeløv, J. K. (2019). PsychoPy2: Experiments in behavior made easy. *Behavior Research Methods*, *51*(1), 195–203. 10.3758/s13428-018-01193-y 30734206 10.3758/s13428-018-01193-y PMC6420413

[CR43] Pu, Y., Kong, X. Z., Ranganath, C., & Melloni, L. (2022). Event boundaries shape temporal organization of memory by resetting temporal context. *Nature Communications*, *13*(1), 1–13. 10.1038/s41467-022-28216-9 10.1038/s41467-022-28216-9 PMC8810807 35110527

[CR44] Radvansky, G. A., & Zacks, J. M. (2017). Event boundaries in memory and cognition. *Current Opinion in Behavioral Sciences,**17*, 133–140. 10.1016/j.cobeha.2017.08.006 29270446 10.1016/j.cobeha.2017.08.006 PMC5734104

[CR45] Richmond, L. L., Gold, D. A., & Zacks, J. M. (2017). Event perception: Translations and applications. *Journal of Applied Research in Memory and Cognition,**6*(2), 111–120. 10.1016/j.jarmac.2016.11.002 28936393 10.1016/j.jarmac.2016.11.002 PMC5602591

[CR46] Ryan, J., & Rogers, M. (2021). Event segmentation deficits in ADHD. *Journal of Attention Disorders,**25*(3), 355–363. 10.1177/1087054718799929 30205738 10.1177/1087054718799929

[CR47] Ryan, J., Burr, Z., Rogers, M. A., & Coplan, R. J. (2023). ADHD and anxiety symptom comorbidity from an event segmentation lens. *Journal of Experimental Psychopathology*. 10.1177/20438087231204168

[CR48] Sargent, J. Q., Zacks, J. M., Hambrick, D. Z., Zacks, R. T., Kurby, C. A., Bailey, H. R., Eisenberg, M. L., & Beck, T. M. (2013). Event segmentation ability uniquely predicts event memory. *Cognition,**129*(2), 241–255. 10.1016/j.cognition.2013.07.002 23942350 10.1016/j.cognition.2013.07.002 PMC3821069

[CR49] Sasmita, K., & Swallow, K. M. (2023). Measuring event segmentation: An investigation into the stability of event boundary agreement across groups. *Behavior Research Methods*, *55*(1), 428–447. 10.3758/s13428-022-01832-5 35441362 10.3758/s13428-022-01832-5 PMC9017965

[CR50] Schoenenkorb, C., Niekerken, L., Valente, G., De Weerd, P., & van de Ven, V. (2023). Temporal memory compression after boundary segmentation and narrative learning. *PsyArxiv*. 10.21608/pshj.2022.250026

[CR51] Schwan, S., & Garsoffky, B. (2004). The cognitive representation of filmic event summaries. *Applied Cognitive Psychology*, *18*(1), 37–55. 10.1002/acp.940

[CR52] Skodzik, T., Holling, H., & Pedersen, A. (2017). Long-term memory performance in adult ADHD: A meta-analysis. *Journal of Attention Disorders*, *21*(4), 267–283. 10.1177/1087054713510561 24232170 10.1177/1087054713510561

[CR53] Skowronek, J. S., Leichtman, M. D., & Pillemer, D. B. (2008). Long-term episodic memory in children with Attention-Deficit/Hyperactivity disorder. *Learning Disabilities Research & Practice,**23*(1), 25–35. 10.1111/j.1540-5826.2007.00260.x

[CR54] Sonuga-Barke, E. J. S., & Castellanos, F. X. (2007). Spontaneous attentional fluctuations in impaired states and pathological conditions: A neurobiological hypothesis. *Neuroscience and Biobehavioral Reviews*, *31*(7), 977–986. 10.1016/j.neubiorev.2007.02.005 17445893 10.1016/j.neubiorev.2007.02.005

[CR55] van de Ven, V., Jäckels, M., & De Weerd, P. (2022). Time changes: Timing contexts support event segmentation in associative memory. *Psychonomic Bulletin & Review,**29*, 568–580. 10.3758/s13423-021-02000-0 34647275 10.3758/s13423-021-02000-0 PMC9038903

[CR56] van de Ven, V., Kleuters, G., & Stuiver, J. (2023). Multisensory synchrony of contextual boundaries affects temporal order memory, but not encoding or recognition. *Psychological Research,**87*, 583–597. 10.1007/s00426-022-01682-y 35482089 10.1007/s00426-022-01682-y PMC9047581

[CR57] van Helvoort, D., Stobbe, E., Benning, R., Otgaar, H., & van de Ven, V. (2020). Physical exploration of a virtual reality environment: Effects on spatiotemporal associative recognition of episodic memory. *Memory & Cognition,**48*(5), 691–703. 10.3758/s13421-020-01024-6 32103427 10.3758/s13421-020-01024-6 PMC7320060

[CR58] Willcutt, E. G. (2012). The prevalence of DSM-IV Attention-Deficit/Hyperactivity disorder: A meta-analytic review. *Neurotherapeutics,**9*(3), 490–499. 10.1007/s13311-012-0135-8 22976615 10.1007/s13311-012-0135-8 PMC3441936

[CR59] Willcutt, E. G. (2023). Attention-Deficit/Hyperactivity Disorder. In D. J. C. Herrmann, E. A. Phelps, & D. S. Gazzaniga (Eds.), *APA Handbook of Neuropsychology* (pp. 523–546). American Psychological Association. 10.1016/B978-0-443-11724-4.00115-0

[CR60] Willems, R. M., Nastase, S. A., & Milivojevic, B. (2020). Narratives for neuroscience. *Trends in Neurosciences,**43*(5), 271–273. 10.1016/j.tins.2020.03.003 32353331 10.1016/j.tins.2020.03.003

[CR61] Woods, K. J. P., Siegel, M. H., Traer, J., & McDermott, J. H. (2017). Headphone screening to facilitate web-based auditory experiments. *Attention, Perception & Psychophysics,**79*(7), 2064–2072. 10.3758/s13414-017-1361-2 10.3758/s13414-017-1361-2 PMC5693749 28695541

[CR62] Yeshurun, Y., Swanson, S., Simony, E., Chen, J., Lazaridi, C., Honey, C. J., & Hasson, U. (2017). Same Story, Different Story: The Neural Representation of Interpretive Frameworks. *Psychological Science*, *28*(3), 307–319. 10.1177/0956797616682029 28099068 10.1177/0956797616682029 PMC5348256

[CR63] Zacks, J. M. (2020). Event Perception and Memory. *Annual Review of Psychology*, *71*, 165–191. 10.1146/annurev-psych-010419-051101 31905113 10.1146/annurev-psych-010419-051101 PMC8679009

[CR64] Zacks, J. M., Braver, T. S., Sheridan, M. A., Donaldson, D. I., Snyder, A. Z., Ollinger, J. M., Buckner, R. L., & Raichle, M. E. (2001a). Human Brain Activity Time-Locked. *Nature Neuroscience*, *4*(6), 651–655. http://neurosci.nature.com 11369948 10.1038/88486

[CR65] Zacks, J. M., Tversky, B., & Iyer, G. (2001b). Perceiving, remembering, and communicating structure in events. *Journal of Experimental Psychology: General*, *130*(1), 29–58. 10.1037/0096-3445.130.1.29 11293458 10.1037/0096-3445.130.1.29

[CR67] Zhao, X., Li, H., Wang, E., Luo, X., Han, C., Cao, Q., Liu, L., Chen, J., Wang, C., Johnstone, S. J., Wang, Y., & Sun, L. (2020). Neural Correlates of Working Memory Deficits in Different Adult Outcomes of ADHD: An Event-Related Potential Study. *Frontiers in Psychiatry*, *11*(May), 1–11. 10.3389/fpsyt.2020.00348 32425833 10.3389/fpsyt.2020.00348 PMC7206828

[CR68] Zheng, Q., Wang, X., Chiu, K. Y., & Shum, K. K. (2022). Time perception deficits in children and adolescents with ADHD: A meta-analysis. *Journal of Attention Disorders,**26*(2), 267–281. 10.1177/1087054720978557 33302769 10.1177/1087054720978557

